# Staphylococcal Infections in Children, California, USA, 1985–2009

**DOI:** 10.3201/eid1901.111740

**Published:** 2013-01

**Authors:** Kathleen Gutierrez, Meira S. Halpern, Clea Sarnquist, Shila Soni, Anna Chen Arroyo, Yvonne Maldonado

**Affiliations:** Author affiliations: Stanford University School of Medicine, Stanford, California, USA

**Keywords:** staphylococcal infection, methicillin-resistant Staphylococcus aureus, MRSA, epidemiology, pediatrics, children, California, bacteria, antimicrobial resistance, staphylococci

## Abstract

Young children, Black children, and those without private insurance were at higher risk for hospitalization.

## Medscape CME Activity

Medscape, LLC is pleased to provide online continuing medical education (CME) for this journal article, allowing clinicians the opportunity to earn CME credit. 

This activity has been planned and implemented in accordance with the Essential Areas and policies of the Accreditation Council for Continuing Medical Education through the joint sponsorship of Medscape, LLC and Emerging Infectious Diseases. Medscape, LLC is accredited by the ACCME to provide continuing medical education for physicians.

Medscape, LLC designates this Journal-based CME activity for a maximum of 1 *AMA PRA Category 1 Credit(s)*^TM^. Physicians should claim only the credit commensurate with the extent of their participation in the activity.

All other clinicians completing this activity will be issued a certificate of participation. To participate in this journal CME activity: (1) review the learning objectives and author disclosures; (2) study the education content; (3) take the post-test with a 70% minimum passing score and complete the evaluation at www.medscape.org/journal/eid; (4) view/print certificate.

**Release date: December 12, 2012; Expiration date: December 12, 2013**

## Learning Objectives

Upon completion of this activity, participants will be able to:

- Assess temporal trends of staphylococcal infection among hospitalized children
- Distinguish risk factors for staphylococcal infection among hospitalized children
- Analyze clinical characteristics of staphylococcal infection among hospitalized children
- Evaluate the microbiology of staphylococcal infection among hospitalized children.

## CME Editor

**Claudia Chesley, **Technical Writer/Editor, Emerging Infectious Diseases. *Disclosure: Claudia Chesley has disclosed no relevant financial relationships*.

## CME Author

**Charles P. Vega, MD, **Health Sciences Clinical Professor; Residency Director, Department of Family Medicine, University of California, Irvine. *Disclosure: Charles P. Vega, MD, has disclosed no relevant financial relationships.*

## Authors

*Disclosures: ****Kathleen Gutierrez, MD****, has disclosed the following relevant financial relationships: served as an advisor or consultant for Cerexa. *Meira S. Halpern, PhD*; ****Clea Sarnquist, DrPH, MPH****; ****Shila Soni, MS****; and ****Anna Chen Arroyo, MD, PhD****, have disclosed no relevant financial relationships. ****Yvonne Maldonado, MD****, has disclosed the following relevant financial relationships: served as an advisor or consultant for DMC, Pfizer; received grants for clinical research from National Institutes of Health, Centers for Disease Control and Prevention, World Health Organization, State of California.*
