# Supplementary material for: Staphylococcal Infections in Children, California, USA, 1985–2009
Source: Emerg Infect Dis. 2013 Jan;19(1):10–20. doi: 10.3201/eid1901.111740 (PMC3557972; doi:10.3201/eid1901.111740)
Supplement: Technical Appendix — International Classification of Diseases, Ninth Revision, Clinical Modification/Diagnosis Related Group codes and infection categories; definitions used to determine the time of infection onset; and a description of an extrapolation scheme used in a study to determine the incidence of and trends in staphylococcal infection–associated hospitalizations among children, California, 1985–2009. [file 11-1740-Techapp-s1.pdf]

Article DOI: http://dx.doi.org/10.3201/eid1901.111740

Staphylococcal Infections in Children, California, 1985–2009

Technical Appendix

Technical Appendix Table 1. Staphylococcal infection–related ICD-9-CM and DRG codes used*

ICD-9-CM and DRG code(s)	Diagnosis
ICD-9-CM	
041.1	Staphylococcal infection
041.11without V09.0	MSSA other
041.11 plus V09.0 or 041.12	MRSA other
038.1	Staphylococcal septicemia
038.11 without V09.0	MSSA septicemia
038.11 plus V09.0 or 038.12	MRSA septicemia
482.4	Staphylococcal pneumonia
482.41without V09.0	MSSA pneumonia
482.41 plus V09.0 or 482.42	MRSA pneumonia
DRG	
DRG 279 (through 2007); MS-DRG 602–603 (2008 forward)	Cellulitis
DRG 385–390 (through 2007); MS-DRG 789–390 (2008 forward)	Neonatal hospitalizations, except for normal newborn
**Coding chronology**	**Change**
Year	
1985–1992	No differentiation between *S. aureus* and other staphylococci, 4-digit codes only
1992, fourth quarter	041.1, a fifth digit was added to specify type of infection: 0, unspecified; 1, *S. aureus*; 9, other
1993	V09.0: Penicillin resistance
1996	A “present on admission code” was added
1997, fourth quarter	038.1, a fifth digit was added to specify type of infection: 0, unspecified; 1, *S. aureus*; 9, other
1998, fourth quarter	482.4, a fifth digit was added to specify type of infection: 0, unspecified; 1, *S. aureus*; 9, other
2003	V09.9 also used to code for MRSA
2008	DRG was replaced by MS-DRG
2008, fourth quarter	MRSA 038.1, 041.1, and 482.4, a fifth digit (2) was added to indicate MRSA

*ICD-9-CM, International Classification of Diseases, Ninth Revision Clinical Modification; DRG, Diagnosis Related Group; MS-DRG, Medicare-Severity Diagnosis Related Group (replaced DRG for discharges starting on January 1, 2008); MSSA, methicillin-susceptible *S. aureus*; MRSA, methicillin-resistant *S. aureus*.

Technical Appendix Table 2. Definitions used to determine the source of infection*

Code	Used if
CO	The code for staphylococcal infection was POA
CO-HCA	The code for staphylococcal infection was POA, plus at least one of the following: 1) evidence of previous treatment, such as the presence of a central venous catheter, dialysis, or surgery; 2) evidence of complications of previous medical treatments; 3) history of a transplanted organ; 4) diagnosis of immune deficiency, cancer, or severe chronic illness; or 5) transfer from acute-care or medium- or low-care facilities
HO	The code for staphylococcal infection was not POA; this is the best estimate of hospital-onset of infection because the dataset did not indicate at what point during hospitalization infection was identified

*Definitions were based on the present-on-admission code; records missing this code were excluded from source of infection analyses. CO, community onset; POA, present on admission; CO-HCA, community onset health–care associated; HO, hospital onset.

Extrapolation Scheme: Estimating the 'True' Values of Number of Admissions (NOA) and Length of Stays in the Hospital (LOS) for the year 2009

The records for admissions with a particular condition during 1985–2008 were used to assess 1) the number and 2) the mean LOS of admissions with that condition during 2009. The following variables are used throughout this appendix:

NOA—yearly number of admissions.

YEC-NOA—end of year censored NOA: number admitted and discharged in the same year.

Est-NOA—estimated value of NOA predicted by the design scheme.

Mean-LOS—mean LOS for all NOA admissions for the year.

YEC-LOS—mean LOS of the records counted in YEC-NOA.

Est-LOS—estimated mean-LOS predicted by the design scheme.

For 1985–2008, all admissions are known. NOA (mean-LOS) for these years were regressed against the year of admission (YOA), YEC-NOA and YEC-LOS, to obtain the parameters later used in calculating Est-NOA (Est-LOS) for each of 1985–2009 in which the codes for the conditions of interest were already introduced. We used the backwards elimination scheme, with stay criterion $p<0.05$. If the p-value for the intercept was <0.05 then the regression model was rerun without intercept.

In order that the covariates in the models be of the similar order of magnitude, the following transformations were used:

NOA (YEC-NOA)—scaled so that it is between 1 and 10.

Mean-LOS (YEC-LOS)—centered around the middle of the interquartile interval of LOS

for all of the 1985–2008 admissions.

YOA—presented as number of years from 1985.

To assess the fitness of the model, we calculated Est-NOA (Est-LOS), using the model chosen by the regression scheme, for each of the years 1985–2008, and the

relative error = 100(NOA − Est-NOA)/NOA [100(mean-LOS − Est-LOS)/mean-LOS]

was calculated for each year. The mean of the relative error of Est-NOA and Est-LOS for staphylococcal infection in the years 1985–2008 were 0.3% (±<0.1%) and 1.7% (±0.3%), respectively. More details are available from the authors.
