# Supplementary material for: Staphylococcal Infections in Children, California, USA, 1985–2009
Source: Emerg Infect Dis. 2013 Jan;19(1):10–20. doi: 10.3201/eid1901.111740 (PMC3557972; doi:10.3201/eid1901.111740)
Supplement: Related texts

# Research article: Staphylococcal Infections in Children, California, USA, 1985–2009

**Authors:** Kathleen Gutierrez, Meira S. Halpern, Clea Sarnquist, Shila Soni, Anna Chen Arroyo, Yvonne Maldonado

**Affiliations:** Author affiliations: Stanford University School of Medicine, Stanford, California, USA

## Abstract

We conducted a retrospective, observational, population-based study to investigate the effect of staphylococcal infections on the hospitalization of children in California during 1985–2009. Hospitalized children with staphylococcal infections were identified through the California Office of Statewide Health Planning and Development discharge database. Infections were categorized as community onset, community onset health care–associated, or hospital onset. Infection incidence was calculated relative to all children and to those hospitalized in acute-care facilities. A total of 140,265 records were analyzed. Overall incidence increased from 49/100,000 population in 1985 to a peak of 83/100,000 in 2006 and dropped to 73/100,000 in 2009. Staphylococcal infections were associated with longer hospital stays and higher risk for death relative to all-cause hospitalizations of children. The number of methicillin-resistant *Staphylococcus aureus* infections increased, and the number of methicillin-susceptible *S. aureus* infections remained unchanged. Children <3 years of age, Blacks, and those without private insurance were at higher risk for hospitalization.

The increasing prevalence of antimicrobial drug–resistant staphylococcal infections is a threat to public health in the United States. Methicillin-resistant *Staphylococcus aureus* (MRSA) is now a frequent cause of skin, soft tissue, and invasive *S. aureus* infections requiring hospitalization in the United States (*1**–**6*).

The characteristics of staphylococcal infection in children in particular communities have been described (*7*–*9*). However, there are fewer population-based studies, and little is known about changes in MRSA transmission among children in California. We conducted a retrospective observational study to determine the incidence of and trends in staphylococcal infection–associated hospitalizations among children in California during 1985–2009.

## Methods

### Study Population

The California Office of Statewide Health Planning and Development (OSHPD) maintains a hospital discharge database that includes demographic and clinical information on patients discharged from hospitals. In compliance with the California Health Safety Code (Section 128735), nonfederal hospitals in the state report all patient discharges to OSHPD. We extracted records from the OSHPD database for children (persons <18 years of age) who were admitted to and discharged from an acute-care facility during January 1, 1985–December 31, 2009, with a primary diagnosis of staphylococcal infection or 1 of <24 other diagnoses. To reduce multiple counting of the same event, we excluded data for children transferred to another acute-care facility within 2 days of admission. International Classification of Diseases, Ninth Revision (ICD-9), Clinical Modification or Diagnosis Related Group (DRG), codes were used to extract medical records and to further classify extracted records (Technical Appendix Table 1). Infections were categorized as community onset (CO), CO health care–associated (CO-HCA), or hospital onset (HO) (Technical Appendix Table 2). Information obtained included age, sex, race, hospital length of stay (LOS), expected source of payment, diagnosis, and medical procedures. Age-related data were analyzed by age group (Table 1). We refer to babies hospitalized at <30 days of age as neonates, independent of the age at which they received a diagnosis of staphylococcal infection or were discharged. No attempt was made to identify patients or access their medical records.

**Table 1:** Incidence of and length of stay for hospitalized children with staphylococcal infections, California, USA, 1985–2009*

Sociodemographic variable and infection category	% Total population of children, N = 140,265	Incidence of children hospitalized among		LOS, d, median (IQR)	% Children who died†
		General population, per 100,000 children	Hospitalized children, per 1,000 children		
Overall	100	62	14	7 (4–21)	2.5
Sex					
M	57	69	15	7 (4–21)	2.5
F	43	56	14	7 (4–21)	2.6
Race					
White	37	60	13	7 (3–18)	2.3
Black	10	88	16	8 (4–25)	2.9
Hispanic	42	63	15	7 (4–21)	2.4
Asian	6	40	13	7 (4–21)	2.9
Age at admission					
<30 d (neonates)	25	452‡	8	28 (7–69)	4.5
31–91 d	6		21	7 (4–17)	3.2
92–365 d	12		21	6 (3–13)	2.3
1–2 y	15	83	22	5 (3–10)	1.5
3–5 y	9	34	17	6 (3–13)	1.5
6–9 y	9	26	17	6 (3–13)	1.5
10–13 y	10	28	19	7 (4–14)	1.6
14–17 y	14	40	16	6 (3–14)	2.0
Expected source of payment					
Private insurance	41	NA	12	7 (3–20)	2.4
Other§	59	NA	16	8 (4–21)	2.6
Staphylococcal infection category					
Uncomplicated¶	72	45	10	6 (3–14)	1.3
Pneumonia only	6	4	1	14 (7–37)	5.7
Septicemia only	20	12	3	14 (6–45)	5.5
Complicated#	2	1	<0.5	38 (16–92)	7.3

*LOS, length of stay; IQR, interquartile range; NA, not applicable. †For differences >0.2% in the proportion of children that died, p<0.0001 (χ^2^ test); p = 0.04 for the differences in the proportion of deaths for Hispanic vs. White children. ‡Combined value for all children <1 year of age. §Medi-Cal, Medicare, other government sources, charity, and none. ¶No code for septicemia or pneumonia identified. #>2 staphylococcal codes identified, including >1 code for septicemia or pneumonia.

**Table 2:** Risk of staphylococcal infection, by demographic characteristic, for children, California, 1985–2009*

Demographic characteristics	Risk of infection, OR† (95% CI)	
	General population of children	Hospitalized children
Sex		
M	1.25 (1.23–1.26)	1.07 (1.06–1.09)
F	Reference	Reference
Race		
White	Reference	Reference
Black	1.46 (1.43–1.49)	1.05 (1.04–1.07)
Hispanic	0.94 (0.93–0.95)	0.96 (0.95–0.97)
Asian	0.63 (0.62–0.65)	Dropped
Age at admission		
<30 d	5.64 (5.55–5.73)‡	0.39 (0.39–0.40)
31–91 d		0.95 (0.92–0.97)
92–365 d		0.98 (0.96–1.00)
1–2 y	Reference	Reference
3–5 y	0.42 (0.41–0.43)	0.80 (0.78–0.81)
6–9 y	0.31 (0.31–0.32)	0.81 (0.79–0.92)
10–13 y	0.34 (0.33–0.35)	0.90 (0.88–0.92)
14–17 y	0.48 (0.47–0.49)	0.76 (0.74–0.77)
Expected source of payment		
Private insurance	NA	0.78 (0.77–0.79)
Other§	NA	Reference

*OR, odds ratio; NA, not applicable. †ORs were calculated by using backward logistic regression (stay criteria p<0.01) with the variables listed in the table and year of admission.  ‡Combined value for all children <1 year of age. §Medi-Cal, Medicare, other government sources, charity, and none.

### Race

Until 1994, race was specified in OSHPD data as White, Black, Hispanic, Native American, Asian (including Pacific Islanders), Other, or Unknown. Starting in 1995, Hispanic was removed from the options for race, and an ethnicity field, including the following options, was introduced: Hispanic, Non-Hispanic, and Unknown. Because the ethnicity field was not available during the entire study period, we combined race and ethnicity into 5 race categories: White, Black, Hispanic, Asian, and Other. The Hispanic category included all OSHPD data for patients with race or ethnicity identified as Hispanic. Unlike the OSHPD database, for which ≈5% of the records specify Other/Unknown race, the US Census Bureau does not allow for Other/Unknown race. Thus, when estimating incidence rates by race, we did not include data for patients with race identified as Other.

### Social Status

The only OSHPD variable associated with socioeconomic status was expected source of pay. Thus, data for this variable were combined in a 2-level variable (private insurance, yes/no) and used as a surrogate for social status.

### Statistical Analysis

The incidence of staphylococcal infection was calculated relative to estimates of the population of children in California (obtained from the California Department of Finance) and to the number of children admitted to California acute-care facilities during 1985–2009, not including children who were transferred to a different acute-care facility within 2 days of admission or children admitted with codes for nonpathological diagnoses, indicating normal newborns or deliveries. Admission years were considered from January through December. Children hospitalized in 2009 and discharged in 2010 are not included in the database, thus biasing downward the estimates of incidence of disease and mean LOS for 2009. To account for this bias, we designed an extrapolation process (Technical Appendix), which was used in determining all 2009 estimates.

We analyzed 4 primary outcomes: 1) risk for hospitalization with a staphylococcal infection; 2) risk that hospitalized children would be diagnosed with a staphylococcal infection; 3) LOS; and 4) risk for death in children with a diagnosis of staphylococcal infection. Risks were estimated separately for each independent variable and by multivariable logistic regression with the covariates sex; age at admission; race; insurance status; year of admission; and, in the case of death, LOS and complexity of the disease (Tables 1, 2). For the population-based logistic regression, a yes/no “hospitalized with staphylococcal infection” outcome variable was artificially introduced. The value “yes” was assigned by year and demographic attribute to the number of children hospitalized for staphylococcal infection; the value “no” was assigned to the remaining children (e.g., in 1985, a total of 449 White boys <1 year old were assigned the value “yes,” and the other White boys <1 year old were assigned the value “no”). 

Because of the size of the study population, even small differences are highly statistically significant. Therefore, we omitted most p values from the text. We used SAS version 9.1 (SAS Institute, Inc., Cary, NC, USA) to perform statistical analyses.

## Results

Discharge records for 140,265 children in California satisfied inclusion criteria for the study. During 1985–2009, the overall incidence of staphylococcal infections among children in California was 63 cases/100,000 children; incidence was calculated as the mean of the yearly incidences weighted by the yearly population size. Yearly incidences ranged from 49 cases/100,000 children in 1985 to a high of 83/100,000 in 2006, followed by a decrease to 73/100,000 in 2009. The increased incidence of hospitalized children with staphylococcal infections during 2002–2006 mirrored an increase in the number of hospitalized children with cellulitis. The decreased incidence of hospitalizations for cellulitis in subsequent years lagged after the decrease in the incidence of hospitalizations for staphylococcal infections (Figure 1). During 1985–2004, the incidence of all-cause hospitalizations among children decreased; incidence has increased only slightly since then (Figure 1). This decrease resulted in even sharper increases in the incidence of staphylococcal infections among hospitalized children (from 8.2 cases/1,000 children in 1985 to 23.3/1,000 in 2006, decreasing to 19.9/1,000 in 2009).

**Figure 1:** Hospitalization trends for children <17 years of age, California, USA, 1985–2009. The incidence of hospitalizations and mean length of stay for children with staphylococcal infection (SI) are compared with the incidences of hospitalizations for cellulitis (Diagnosis Related Group [DRG] 279 or Medicare Severity–DRG 602–603) and for all-cause hospitalizations of children. The horizontal line separates the incidence graphs, which are to be read against the left axis, and the graph for length of stay, which is read against the right axis.

Demographic information for hospitalized children with staphylococcal infection is shown in Figure 2. All groups except neonates demonstrated an increase in infection during 2002–2006. The increase was most pronounced in White and Black children (60%); the smallest increase was among Asian children (40%).

**Figure 2:** Hospitalization trends for children <17 years of age with staphylococcal infection, California, USA, 1985–2009. Data are no. of patients/100,000 population, except as indicated for children <1 year of age. A) Trends by sex. B) Trends by race. C) Trends by age group, age >1 year. D) Trends for infants (children <364 days of age) compared with trends for children 1–17 years of age; *number/100,000 children <1 year of age.

Table 1 shows the incidence of staphylococcal infection, by demographic characteristic, among the population and among hospitalized children; Table 2 shows the odds ratios (ORs) for the risk of infection estimated while controlling for year of admission and demographic characteristics. Compared with children 1–2 years of age, those <1 year of age were at high risk for hospitalization with staphylococcal infection (OR 5.6) (Table 2). Black children were 1.5× more likely and Asian children 0.6× less likely than White children to be hospitalized with staphylococcal infection (Tables 1, 2). However, among hospitalized children, the risk of acquiring staphylococcal infection was low for neonates compared with children 1–2 years of age (OR 0.4); for infants 1–12 months of age, the risk was approximately equal to that for older children. Lack of private insurance was the single most dominant factor associated with increased risk for staphylococcal infection among hospitalized children (OR 1.3) (Table 2); the association with race was much lower (ORs of 1.05, 0.96, and 1.0 for Black, Hispanic, and Asian race, respectively, compared with White race) (Table 2).

### Length of Hospital Stay

Hospital stays ranged from 0 to 2,067 days (median 7 days, mean 20.7 ± 0.1 days); for 10% of the hospitalizations, LOS was >60 days. LOS varied by sociodemographic factor (Table 1). Children hospitalized in the first month of life had long LOSs (median 28 days). Children without private insurance had longer LOSs (median 8 days) than children with private insurance (median 7 days).

Extremely long LOSs were mostly among premature infants. Neonates hospitalized within 2 days of birth made up 16% of the study population, but they represented 65% of the children hospitalized for >3 months. When we excluded neonates from the analysis, LOS was shorter and the 10%–90% interval was 2–31 days (median 6 days, mean 13.9 days). However, even after we excluded neonates from analysis, LOS was still considerably longer for hospitalized children with staphylococcal infections than for children hospitalized for any other cause (median LOS 2 days, mean 4.9 days). During 1985–2002, the mean LOS for hospitalized children with staphylococcal infections increased from 17.3 to 26.7 days, but by 2009, the mean LOS decreased to 17.7 days (Figure 1).

### Outcomes

Among all children with a diagnosis of staphylococcal infection, 82.6% had a routine discharge, 10.4% required additional low- or medium-intensity care on discharge, 4.4% were transferred to another acute care facility, and 2.5% died. Using a multivariable model controlling for year of admission, LOS, age at admission, sex, race, and insurance status, we compared the risk for death among children with and those without a diagnosis of staphylococcal infection: the risk was higher for children with staphylococcal infection (OR 2.1, 95% CI 2.0%–2.2%). Among hospitalized children with staphylococcal infections, the risk for death was associated with the complexity of the disease and with some demographic factors (Table 1). Most relationships remained unchanged in multivariable analysis that included LOS and the factors presented in Table 1. The exceptions were higher risk of death for children of Asian race, but not Hispanic or Black race, compared with White race (OR 1.2, 95% CI 1.1%–1.4%) and not having private insurance (OR 1.6, 95% CI 1.5%–1.9%). For hospitalized children with staphylococcal infections, the risk of death increased from 2.4% in 1985 to 3.5% in 2002; the risk decreased beginning in 2003 and remained at <2% during 2006–2009.

### Temporal Trends in Incidence

Among the entire population of children in California, the yearly incidence of staphylococcal infection increased from 1985 to 2009 (Figure 1). The increase was not uniform: incidence decreased during 1996–2000, followed by a large increase during 2002–2006. Overall, 38% of the records for hospitalized children with staphylococcal infection had an ICD-9-CM code for skin and soft tissue infection (SSTI), and 67% of those records had an ICD-9-CM code for cellulitis. The proportion of hospitalized children with staphylococcal infection who also had an ICD-9-CM code for cellulitis remained at <20% through 2002 and then rose to 44.4% in 2009. The rise in hospitalizations for staphylococcal infections corresponded to a 2002–2007 rise in hospitalizations for cellulitis (from 34 to 56/100,000 children), as classified by DRG (Figure 1). The percentage of records including DRG-classified cellulitis and staphylococcal infection increased from 13%–19% during 1985–2002 to 40%–44% during 2005–2009. During this period, all-cause hospitalizations for children in California did not increase (Figure 1).

The increased incidence of staphylococcal infections during 2002–2005 was seen across all racial and age groups, except neonates. The steepest increase was seen among Black children and children 1 month–2 years of age; the increase was less pronounced among Asian children *(*Figure 2). The rise in incidence among children 1 month–2 years of age was observed in all races.

### Diagnostic Categories of Infections

The increase in the incidence of hospitalizations for staphylococcal infections, which began in 2002, was driven by diagnoses of MRSA infection and cellulitis (Figure 3). In 1994, codes were introduced to distinguish between MRSA and methicillin-sensitive *S. aureus* (MSSA) infections (Technical Appendix). During the late 1990s, as the proportion of identified infections increased, the proportion of records with infections of unknown type dropped sharply (Figure 3, panel B). During 2000 and 2009, >90% of the hospitalization records identified an infection type: the proportion of MSSA infections decreased, and the incidence of MRSA-related hospitalizations increased >10-fold (from 3 to 35/100,000 children). Hospitalizations for MRSA cellulitis accounted for 46% of the MRSA-related hospitalizations; MRSA pneumonia and septicemia accounted for 6% and 4.5%, respectively (Figure 3, panels C–E).

**Figure 3:** Hospitalization trends for children <17 years of age with staphylococcal infection (SI), California, USA, 1985–2009. A) Population incidence of particular SI-associated diagnoses relative to the incidence of the same diagnoses in 2000. B) Percentage of all SI-coded hospitalization records that were further classified by various types of SI; the classification code has been available only since 1994. C) Trends for types of SI with cellulitis; available only since 1994. Data are no. of patients/100,000 population. D) Trends for types of SI with pneumonia; available only since 1999. Data are no. of patients/1,000,000 population. E) Trends for types of SI with septicemia; available only since 1998. Data are no. of patients/1,000,000 population. MSSA, methicillin-susceptible *Staphylococcus aureus*; MRSA, methicillin-resistant *S. aureus*.

### Infections in Neonates versus Older Children

The population incidence of staphylococcal infection–related hospitalizations was higher for neonates than older infants and children (Figure 2, panel D). This incidence is the number of cases of staphylococcal infection in neonates relative to the number of babies born that year; it increases 12-fold if estimated relative to the number of babies who were <1 month old. However, the risk for a staphylococcal infection in all-cause hospitalized neonates was much lower than the risk in all-cause hospitalized older babies (Tables 1, 2). During 2002–2006, a sharp increase in the incidence of staphylococcal infections occurred for all age groups except neonates, and the decrease that occurred in 2007–2009 was faster among neonates than other age groups (Figure 2d). Compared with staphylococcal infections in older children, those in neonates were associated with longer LOSs and poorer clinical outcomes (Table 1) and with reduced likelihood of being caused by *S. aureus* (42% vs. 76%) or MRSA if caused by *S. aureus* (21% vs. 32%). Coagulase-negative staphylococci were assumed to be the cause of some non–*S. aureus* staphylococcus infections.

### Community-onset versus Hospital-onset Infection, 1996–2009

Because of the differences in types of staphylococcal infections (noted above), we excluded infections in neonates from this analysis. The demographics for children with CO, CO-HCA, and HO staphylococcal infections are shown in Table 3.

**Table 3:** Staphylococcal infections in non-neonate children, by onset location, California, 1996–2009*

Variable	All, N = 66,141	CO	CO-HCA	HO
% of all records	100	48	41	11
Overall incidence, no./100,000 population	49	24	20	5
Demographic data				
Sex				
M	56	46	42	12
F	44	51	39	10
Race				
White	34	46	43	10
Black	10	51	39	10
Hispanic	46	48	40	11
Asian	6	49	38	12
Age at admission				
31–91 d	6	52	33	16
92–365 d	16	57	32	11
1–2 y	21	58	34	9
3–5 y	12	41	49	10
6–9 y	12	41	49	10
0–13 y	14	42	47	11
14–17 y	19	43	45	12
Expected source of payment				
Private insurance	39	48	41	11
Other†	61	48	41	11
Outcome				
% Death	1.7	0.1	2.0	7.5
% Normal discharge	84.2	92	81	65
Mean (SE) LOS, d	12.5 (0.1)	5.3 (<0.1)	13.6 (0.1)	40.7 (0.5)
Median (interquartile range) LOS, d	6 (3–13)	4 (3–6)	8 (4–15)	28 (15–51)
Infection category and type				
Staphylococcal infection category				
Uncomplicated‡	79	91	71	61
Pneumonia	6	2	8	11
Septicemia	13	5	19	25
Complicated§	2	1	3	4
Type of staphylococcal infection				
Unknown	11	8	13	14
Other than *Staphylococcus aureus*	19	10	25	37
MSSA	42	43	42	36
MRSA	29	40	20	13

*Data represent the association of particular attributes with the particular locations of infection onset: 1) first column, for demographic attributes and category/type of infection, data are the % of all the records; 2) columns 2–4, for demographic attributes, the data represent the distribution of an attribute among CO, CO-HCA, and HO onset cases; and 3) columns 2–4, for category/type of infection, data are the % of all the records with CO, CO-HCA, or HO onset. CO, community onset; CO-HCA, CO–health care associated; HO, hospital onset; LOS, length of stay in hospital; MSSA, methicillin-sensitive *S. aureus*; MRSA, methicillin-resistant *S. aureus* †Medi-Cal, Medicare, other government sources, charity, and none. ‡No code for septicemia or pneumonia identified. §>2 staphylococcal codes identified, including >1 code for septicemia or pneumonia.

The incidences of hospitalization for CO, CO-HCA, and HO MSSA and MRSA infections are shown in Figure 4, panels A, B. Throughout the study period, ≈10% of MSSA cases were HO infections, and the incidence of CO infections was always higher than that for CO-HCA infections. While the incidence of MSSA infections changed little during 2000–2009, the incidence of MRSA infections increased dramatically. HO MRSA infections peaked in 2005, increasing 4.1-fold from 2000 to 2005; CO-HCA and CO infections peaked in 2006, increasing 5.3- and 27.7-fold, respectively, from 2000 to 2006. Until 2001, CO-HCA infections accounted for >50% of MRSA-related hospitalizations. By 2005, >70% of MRSA-related hospitalizations were CO infections, and only 25% were CO-HCA infections. The proportion of HO MRSA infections dropped from 18% in 1996 to <5% in 2005–2009.

**Figure 4:** Hospitalization trends for children 1 month to 17 years of age with *Staphylococcus aureus* infection, by infection onset, California, USA, 1996–2009. Data are no. of patients/100,000 population. A) Incidence of methicillin-susceptible *S. aureus*. B) Incidence of methicillin-resistant *S. aureus.*

A high proportion of CO MRSA infections was associated with SSTIs (Table 4). CO staphylococcus–related septicemia, pneumonia, and musculoskeletal infections were more often associated with MSSA (Table 4).

**Table 4:** Medical conditions associated with community-onset staphylococcal infections in non-neonate children, California, 1996–2009*

Medical condition	Type of infection†			
	All, N = 31,893	MRSA, n = 12,667	MSSA, n = 13,605	Other, n = 3,073
Skin/soft tissue infection				
Records with diagnosis, %	71	91	66	36
Cellulitis, %	84	90	80	63
Deaths, no.	1	0	1	0
Mean (SE) LOS, d	4.5 (<0.1)	4.3 (<0.1)	4.6 (<0.1)	5.5 (0.2)
Median (IQR) LOS, d	3 (2–5)	3 (2–5)	3 (2–5)	4 (2–6)
Sepsis, shock, or bacteremia				
Records with diagnosis, %	12	4	12	29
Deaths, no.	15	1	6	1
Mean (SE) LOS, d	8.0 (0.2)	10.9 (0.4)	8.3 (0.2)	6.6 (0.3)
Median (IQR) LOS, d	6 (4–9)	8 (5–13)	6 (4–10)	5 (3–7)
Respiratory infection				
Records with diagnosis, %	19	13	18	30
Deaths, no.	31	2	19	1
Mean (SE) LOS, d	7.1 (0.1)	6.9 (0.2)	7.3 (0.2)	6.6 (0.3)
Median (IQR) LOS, d	5 (3–8)	4 (3–7)	5 (3–8)	4 (3–7)
Musculoskeletal infections				
Records with diagnosis, %	12	6	20	6
Deaths, no.	0	0	0	0
Mean (SE) LOS, d	9.0 (0.1)	11.0 (0.4)	8.4 (0.1)	9.4 (0.7)
Median (IQR) LOS, d	7 (4–10)	7 (5–13)	6 (4–9)	6 (4–10)

*MRSA, methicillin-resistant *Staphylococcus aureus*; MSSA, methicillin-sensitive *S. aureus*; LOS, length of stay in hospital; IQR, interquartile range †A particular staphylococcal type could not be determined for 11% of the records; thus, added together, numbers in the MRSA, MSSA, and Other staphylococci groups do not equal the number in All types of staphylococcal infection group. Diagnostic groups are not mutually exclusive; therefore, percentages in columns total >100.

## Discussion

The results of this population-based study, spanning 25 years, show major changes in the incidences and clinical manifestations of hospitalizations related to staphylococcal infection in a large and diverse population of children. The yearly incidence of pediatric hospitalizations for staphylococcal infections remained stable during 1985–2002 and then increased substantially from 2003 forward; the change was driven by an increase in MRSA infections. Other investigators have identified similar trends in smaller numbers of patients over a shorter time (*10*–*12*). The increase in incidence paralleled an increase in admissions for cellulitis and peaked in 2006. The decrease in staphylococcus infection–related hospitalizations among children during 2006–2009 corresponded with a decrease in hospitalizations for MRSA-related cellulitis. California OSHPD data for children and adults confirm that the number of hospitalizations for MRSA infection stabilized during 2005–2007 (*13*); our study shows that the trend is sustained. A recent observational study of Department of Defense healthcare beneficiaries of all ages showed a decline in rates of CO and HO MRSA–related bacteremia and in the proportion of CO MRSA–related SSTIs beginning in 2006 (*14*).

The incidence of admissions of children with staphylococcal infections (MSSA, MRSA, and non–*S. aureus* staphylococci infections) was lower in our study than in other studies (14 cases/1,000 admissions vs. 20.8–35.8/1,000 during 2002–2007 in another study [*10*]). The time covered in our study was the main factor driving the lower overall incidence of infections in hospitalized patients; the very low incidence of MRSA infections before 2002 lowered overall incidence. Other factors that possibly contributed to the differences between study findings include differences in patient populations, differences in *S.* *aureus* colonization and susceptibility patterns, and variability in clinical practices. The OSHPD database includes data for children hospitalized at all nonfederal hospitals in California, few of which are solely for children. Some doctors may have a referral bias and send children with chronic medical conditions and a higher risk of acquiring a staphylococcal infection to a children’s hospital.

In our study population, the incidence of hospitalization with a staphylococcal infection was highest among boys, Black children, and, in particular, children <3 years of age (both in the general and in hospital populations). These groups have been found to be at higher risk for MRSA infections (*15*–*17*). Compared with older children, neonates and infants are at higher risk for serious invasive disease when contracting a bacterial infection and, therefore, are more likely to be hospitalized. This increased likelihood of being hospitalized for a bacterial infection may have contributed to an increased population incidence of staphylococcal infections identified in this younger age group of children. The number of all-cause hospitalizations of neonates was higher than that for children in other age groups, partly explaining the relative decreased hospital incidence of staphylococcal infection in this age group. Compared with their privately insured peers, children without private insurance had a higher incidence of hospitalization for staphylococcal infections, longer LOSs, and higher risk for dying. It is unclear why the incidence of hospitalization was higher and LOS was longer for uninsured children, but similar findings have been noted in other analyses of childhood diseases (*18*).

Children who received a diagnosis of staphylococcal infection had longer LOSs and had a greater risk of dying than those who did not receive a diagnosis of staphylococcal infection; we could not determine what role the infection played in the cause of death or extended LOS. In a previous study, we found that the risk for acquiring a staphylococcal infection increased incrementally the longer a patient was hospitalized (*19*); this finding likely reflects the key role that underlying medical conditions have in increasing LOS and risk for death. Among non-neonate children, the highest risk for death in children with staphylococcal infection was in those with HO infection (7.5%); children with CO-HCA and CO staphylococcal infections had a 2.0% and 0.01% risk, respectively. The percentage of death in non-neonates hospitalized with a staphylococcal infection peaked at 2.5% in 2002 and decreased to 1.0% in 2009. The decrease may be partly explained by the relative increase of staphylococcal infections that are CO MRSA SSTIs, which carried a very small risk for death, although deaths continued to decrease even when CO MRSA infections started to decline.

More than half of non-neonates with a diagnosis of staphylococcal infection had CO-HCA or HO infections, and most had MSSA infections (42% and 36%, respectively), followed by MRSA infections (20% and 13%, respectively) and infections with other staphylococci (25% and 37%, respectively). The continued predominance of invasive MSSA infections among children with CO-HCA conditions has been confirmed by others (*8*). Infections with coagulase-negative staphylococci, common pathogens in patients with health care–related conditions, represent some of the infections caused by other staphylococcal (i.e., non-MRSA and non-MSSA) infections (*20*). Despite an increase in the number of hospitalized children with MRSA pneumonia and septicemia, the overall incidence of MRSA invasive disease was low. Children with CO-HCA or HO infections were more likely to have a diagnosis of invasive disease (pneumonia or septicemia) compared with children with CO disease. This clinical pattern is consistent with previously described patterns (*7*,*16*,*21*,*22*). Most CO non-neonatal hospitalizations in this study were due to SSTIs, and MRSA was coded more frequently than MSSA after 2002.

The incidence of HO staphylococcal infection increased until 2005 and has since stabilized. This finding may reflect implementation of more stringent infection-control procedures, including those in neonatal intensive care units. Other investigators have attributed a recent decrease in the national prevalence of inpatient MRSA infections to enhanced infection-control strategies, including active surveillance testing (*23*).

Limitations of this study include inherent concerns regarding the accuracy of ICD-9 coding (*24*); miscoding could have occurred. It is possible that MRSA infections were incorrectly reported in some cases because the code used to indicate resistance to methicillin can also be used to indicate resistance to other antimicrobial drugs in the penicillin family. As awareness of MRSA infections increased, it is likely that diagnostic testing for MRSA increased, leading to an apparent increase in incidence. We did not review medical records for patients identified with staphylococcal infection, and clinical and microbiologic diagnoses could not be verified. The incidence data presented here represent hospitalizations reported to a large state database, not numbers of individual patients. We attempted to correct for possible overcounting by excluding children transferred from 1 acute care institution to another within 2 days of admission. The incidence in nondecennial years was calculated by using state population estimates, which are subject to a small degree of error, pending revisions based on 2010 census data. 

This analysis represents one of the largest population-based studies of staphylococcal infection–related hospitalizations among children. The substantial increase in childhood hospitalizations for staphylococcal infection in California was driven by an increase in CO MRSA SSTIs. The highest incidence of hospitalizations with staphylococcal infection was among children <3 years of age, Black children, and children without private insurance. We also found that the incidence of hospitalizations with staphylococcal infection declined during the last 3 years of the study, corresponding to a decrease in the number of diagnoses of MRSA SSTIs. The reasons for these declines are unknown, but they could include changes in the epidemiology or strains of circulating S. *aureus* or changes by physicians in the management of uncomplicated MRSA infections. It is also possible that extensive MRSA-related prevention education efforts by local health departments are having an effect. Additional studies are needed to determine whether this trend in declining numbers of hospitalizations with staphylococcal infection will be sustained and to provide a better understanding of the epidemiologic or biologic factors or public health and infection-control interventions that may have contributed to the decline in MRSA hospitalizations.

Technical Appendix International Classification of Diseases, Ninth Revision, Clinical Modification/Diagnosis Related Group codes and infection categories; definitions used to determine the time of infection onset; and a description of an extrapolation scheme used in a study to determine the incidence of and trends in staphylococcal infection–associated hospitalizations among children, California, 1985–2009.

## References

[1] King MD, Humphrey BJ, Wang YF, Kourbatova EV, Ray SM, Blumberg HM. Emergence of community-acquired methicillin-resistant *Staphylococcus aureus* USA 300 clone as the predominant cause of skin and soft-tissue infections. Ann Intern Med. 2006;144:309–17 .1652047110.7326/0003-4819-144-5-200603070-00005

[2] Carleton HA, Diep BA, Charlebois ED, Sensabaugh GF, Perdreau-Remington F. Community-adapted methicillin-resistant *Staphylococcus aureus* (MRSA): population dynamics of an expanding community reservoir of MRSA. J Infect Dis. 2004;190:1730–8. 10.1086/42501915499526

[3] Moran GJ, Amii RN, Abrahamian FM, Talan DA. Methicillin-resistant *Staphylococcus aureus* in community-acquired skin infections. Emerg Infect Dis. 2005;11:928–30. 10.3201/eid1106.04064115963289PMC3367577

[4] Frazee BW, Lynn J, Charlebois ED, Lambert L, Lowery D, Perdreau-Remington F. High prevalence of methicillin-resistant *Staphylococcus aureus* in emergency department skin and soft tissue infections. Ann Emerg Med. 2005;45:311–20. 10.1016/j.annemergmed.2004.10.01115726056

[5] Klein E, Smith DL, Laxminarayan R. Hospitalizations and deaths caused by methicillin-resistant *Staphylococcus aureus*, United States, 1999–2005. Emerg Infect Dis. 2007;13:1840–6. 10.3201/eid1312.07062918258033PMC2876761

[6] Mera RM, Suaya JA, Amrine-Madsen H, Hogea CS, Miller LA, Lu EP, Increasing role of *Staphylococcus aureus* and community-acquired methicillin-resistant *Staphylococcus aureus* infections in the United States: a 10-year trend of replacement and expansion. Microb Drug Resist. 2011;17:321–8. 10.1089/mdr.2010.019321417776

[7] Buckingham SC, McDougal LK, Cathey LD, Comeaux K, Craig AS, Fridkin SK, Emergence of community-associated methicillin-resistant *Staphylococcus aureus* at a Memphis, Tennessee Children's Hospital. Pediatr Infect Dis J. 2004;23:619–24. 10.1097/01.inf.0000131981.67342.c415247599

[8] Hultén KG, Kaplan SL, Gonzalez BE, Hammerman WA, Lamberth LB, Versalovic J, Three-year surveillance of community onset health care–associated *Staphylococcus aureus* infections in children. Pediatr Infect Dis J. 2006;25:349–53. 10.1097/01.inf.0000207404.50143.1e16567988

[9] Hussain FM, Boyle-Vavra S, Bethel CD, Daum RS. Current trends in community-acquired methicillin-resistant *Staphylococcus aureus* at a tertiary care pediatric facility. Pediatr Infect Dis J. 2000;19:1163–6. 10.1097/00006454-200012000-0000911144377

[10] Gerber JS, Coffin SE, Smathers SA, Zaoutis TE. Trends in the incidence of methicillin-resistant *Staphylococcus aureus* infection in children's hospitals in the United States. Clin Infect Dis. 2009;49:65–71. 10.1086/59934819463065PMC2897056

[11] Hota B, Ellenbogen C, Hayden MK, Aroutcheva A, Rice TW, Weinstein RA. Community-associated methicillin-resistant *Staphylococcus aureus* skin and soft tissue infections at a public hospital: do public housing and incarceration amplify transmission? Arch Intern Med. 2007;167:1026–33. 10.1001/archinte.167.10.102617533205

[12] Suryadevara M, Moro MR, Rosenbaum PF, Kiska D, Riddell S, Weiner LB, Incidence of invasive community-onset *Staphylococcus aureus* infections in children in Central New York. J Pediatr. 2010;156:152–4.e151. 10.1016/j.jpeds.2009.07.02020006767

[13] California Office of Statewide Health Planning and Development, Healthcare Information Division. OSHPD Health Facts. Methicillin-resistant *Staphylococcus aureus* infections in California hospital patients, 1999 to 2007. 2009 [cited 2012 Nov 1]. http://www.oshpd.ca.gov/HID/Products/Health_Facts/HealthFacts_MRSA_WEB.pdf

[14] Landrum ML, Neumann C, Cook C, Chukwuma U, Ellis MW, Hospenthal DR, Epdemiology of *Staphylococcus aureus* blood and skin and soft tissue infections in the US military health system, 2005–2010. JAMA. 2012;308:50–9. 10.1001/jama.2012.713922760291

[15] Boucher HW, Corey GR. Epidemiology of methicillin-resistant *Staphylococcus aureus.* Clin Infect Dis. 2008;46(Suppl 5):S344–9. 10.1086/53359018462089

[16] Fridkin SK, Hageman JC, Morrison M, Sanza LT, Como-Sabetti K, Jernigan J, Methicillin-resistant *Staphylococcus aureus* disease in three communities. N Engl J Med. 2005;352:1436–44. 10.1056/NEJMoa04325215814879

[17] Klevens RM, Morrison MA, Nadle J, Petit S, Gershman K, Ray S, Invasive methicillin-resistant *Staphylococcus aureus* infections in the United States. JAMA. 2007;298:1763–71. 10.1001/jama.298.15.176317940231

[18] Standridge SM, Horn PS. Variations in hospitalization outcomes in children admitted with seizures between 2003 and 2006. J Child Neurol. 2012;27:898–906. 10.1177/088307381143632522408142

[19] Burke RE, Halpern MS, Baron EJ, Gutierrez K. Pediatric and neonatal *Staphylococcus aureus* bacteremia: epidemiology, risk factors, and outcome. Infect Control Hosp Epidemiol. 2009;30:636–44. 10.1086/59752119496643

[20] Kang J, Sickbert-Bennett EE, Brown V, Weber DJ, Rutala WA. Relative frequency of health care–associated pathogens by infection site at a university hospital from 1980 to 2008. Am J Infect Control. 2012;40:416–20. 10.1016/j.ajic.2011.06.01321943832

[21] Dietrich DW, Auld DB, Mermel LA. Community-acquired methicillin-resistant *Staphylococcus aureus* in southern New England children. Pediatrics. 2004;113:e347–52. 10.1542/peds.113.4.e34715060266

[22] Purcell K, Fergie J. Epidemic of community-acquired methicillin-resistant *Staphylococcus aureus* infections: a 14-year study at Driscoll Children's Hospital. Arch Pediatr Adolesc Med. 2005;159:980–5. 10.1001/archpedi.159.10.98016203945

[23] Jarvis WR, Jarvis AA, Chinn RY. National prevalence of methicillin-resistant *Staphylococcus aureus* in inpatients at United States health care facilities, 2010. Am J Infect Control. 2012;40:194–200. 10.1016/j.ajic.2012.02.00122440670

[24] Ollendorf DA, Fendrick AM, Massey K, Williams GR, Oster G. Is sepsis accurately coded on hospital bills? Value Health. 2002;5:79–81. 10.1046/j.1524-4733.2002.52013.x11918823

# Research article: Staphylococcal Infections in Children, California, USA, 1985–2009

**Authors:** Kathleen Gutierrez, Meira S. Halpern, Clea Sarnquist, Shila Soni, Anna Chen Arroyo, Yvonne Maldonado

**Affiliations:** Author affiliations: Stanford University School of Medicine, Stanford, California, USA

## Medscape CME Quiz

## Earning CME Credit

To obtain credit, you should first read the journal article. After reading the article, you should be able to answer the following, related, multiple-choice questions. To complete the questions (with a minimum 70% passing score) and earn continuing medical education (CME) credit, please go to www.medscape.org/journal/eid. Credit cannot be obtained for tests completed on paper, although you may use the worksheet below to keep a record of your answers. You must be a registered user on Medscape.org. If you are not registered on Medscape.org, please click on the New Users: Free Registration link on the left hand side of the website to register. Only one answer is correct for each question. Once you successfully answer all post-test questions you will be able to view and/or print your certificate. For questions regarding the content of this activity, contact the accredited provider, CME@medscape.net. For technical assistance, contact CME@webmd.net. American Medical Association’s Physician’s Recognition Award (AMA PRA) credits are accepted in the US as evidence of participation in CME activities. For further information on this award, please refer to http://www.ama-assn.org/ama/pub/category/2922.html. The AMA has determined that physicians not licensed in the US who participate in this CME activity are eligible for *AMA PRA Category 1 Credits*™. Through agreements that the AMA has made with agencies in some countries, AMA PRA credit may be acceptable as evidence of participation in CME activities. If you are not licensed in the US, please complete the questions online, print the certificate and present it to your national medical association for review.

## Article Title:  Staphylococcal Infections in Children, California, USA, 1985–2009

## CME Questions

**1. You are caring for an 18-month-old male child admitted with fever, lethargy, and decreased oral intake for 2 days. His blood culture is growing *Staphylococcus aureus*. What should you consider regarding temporal trends of pediatric hospitalizations related to staphylococcal species in the current study?**

A. The biggest increase in the incidence of hospitalizations occurred between 1985 and 2002

B. The biggest increase in the incidence of hospitalizations occurred between 2006 and 2009

C. Hospitalizations for cellulitis declined between 2002 and 2006

D. The maximum incidence of hospitalization remained below 100 cases per 100,000 population

**2. What was one the demographic risk factors for hospitalization with staphylococcal infection in the current study?**

A. Female sex

B. African American race

C. Age 14 to 17 years

D. Low family income

**3. What should you consider regarding outcomes of hospitalization related to staphylococcal infection in the current study?**

A. Excluding neonates, the mean length of hospital stay was approximately 14 days

B. The length of hospital stay progressively increased over the entire study period

C. Staphylococcal infection did not independently increase the risk of mortality

D. African American race was associated with a higher risk of mortality due to staphylococcal infection

**4. What should you consider regarding the types of infection with staphylococcal infections in the current study?**

A. Bacteremia was the most common type of infection

B. Rates of infection with methicillin-susceptible *S. aureus* increased substantially from 2000 to 2009

C. Rates of infection with methicillin-resistant *S. aureus* (MRSA) increased substantially from 2000 to 2009

D. MRSA infections were most common among neonates

## Activity Evaluation

**Table:** 

**1. The activity supported the learning objectives.**				
Strongly Disagree				Strongly Agree
1	2	3	4	5
**2. The material was organized clearly for learning to occur.**				
Strongly Disagree				Strongly Agree
1	2	3	4	5
**3. The content learned from this activity will impact my practice.**				
Strongly Disagree				Strongly Agree
1	2	3	4	5
**4. The activity was presented objectively and free of commercial bias.**				
Strongly Disagree				Strongly Agree
1	2	3	4	5
